# Impact of Health Systems on Life Expectancy in Taiwan, Singapore, and the USA: Similarities and Differences

**DOI:** 10.1093/geroni/igaa057.2475

**Published:** 2020-12-16

**Authors:** Ya-Mei Chen, Yuchi Young, Patrick Schumacher

**Affiliations:** 1 National Taiwan University, Taipei, Taiwan (Republic of China); 2 SUNY at Albany, Rensselaer, New York, United States; 3 University at Albany State University of New York, Rensselaer, New York, United States

## Abstract

Like Singapore, Taiwan has universal health care and universal long-term care (LTC). The USA has neither. Those who need LTC in the US pay out of pocket, buy private LTC insurance or spend down to qualify for Medicaid; there are inherent issues related to cost and quality. For example, nursing home care costs over US$100,000/year and only about 8% of the population has private LTC insurance. Many people become impoverished or struggle financially to qualify for Medicaid. Conversely, Taiwan has universal LTC insurance that offers comprehensive services for all, but it is relatively new and its impact remains to be seen. Similarities and differences related to LTC quality indicators such as functional independence, and the NCQA quality measures (e.g., effectiveness of care, access/availability) in Taiwan, Singapore and the US will be presented. The results of this comparison can inform policy makers and stakeholders leading to best practices. Part of a symposium sponsored by International Comparisons of Healthy Aging Interest Group.

